# Relative effects of four resurgence‐mitigation strategies informed by behavioral momentum theory

**DOI:** 10.1002/jaba.70083

**Published:** 2026-08-23

**Authors:** Daniel R. Mitteer, Wayne W. Fisher, Brian D. Greer, Casey Irwin Helvey

**Affiliations:** ^1^ Children's Specialized Hospital–Rutgers University Center for Autism Research Education, and Services (CSH–RUCARES) Somerset NJ USA; ^2^ Rutgers Robert Wood Johnson Medical School New Brunswick NJ USA; ^3^ Rutgers Brain Health Institute Piscataway NJ USA

**Keywords:** behavioral momentum theory, functional communication training, reinforcement rate, resurgence, treatment duration

## Abstract

Behavioral momentum theory (BMT) posits that particular independent variables may be adjusted during routine differential‐reinforcement treatments (e.g., functional communication training) to reduce resurgence of severe destructive behavior. Across four consecutive studies using similar preparations and participants, our research group investigated four BMT‐informed mitigation strategies: (a) lean reinforcement rates for target behavior during baseline, (b) lean reinforcement rates for alternative behavior during treatment, (c) a longer treatment duration, and (d) a combined approach including all three refinements. In the current study, we analyzed the relevance of each BMT‐informed strategy sourced from four published studies and included three additional participants resulting in a total of 24 data sets. Lean reinforcement rates during baseline and the combined approach resulted in the largest reductions in resurgence when analyzed as a proportion of baseline. We contextualize these findings in relation to BMT's account of resurgence and implications for practice.

The long‐term effectiveness of function‐based interventions for severe destructive behavior (e.g., self‐injury, aggression) requires the continued suppression of destructive behavior and, ideally, the persistence of adaptive responding (e.g., communication, cooperation; Wacker et al., 2011). Unfortunately, challenges to the effectiveness of such interventions (e.g., periods of extinction for the alternative response) are common, and when they occur, prior research has shown that they often disrupt intervention effects, sometimes leading to substantial levels of treatment relapse, or a recurrence of destructive behavior following effective treatment (see top panel of Figure 3 in Mitteer et al., 2022, for one representation). These concerns regarding the durability of function‐based interventions for destructive behavior have led some in the field (e.g., Nevin & Wacker, 2013) to emphasize the importance of systematically arranging the environment to explicitly challenge the efficacy of intervention procedures rather than being content with improvements in behavior occurring only under the idealistic conditions of high‐quality treatment implementation.

Many translational investigators have heeded such calls for new lines of research that seek to better understand the conditions under which treatment effects maintain. Several research teams have investigated the prevalence of different forms of treatment relapse and relapse‐like phenomena in the clinic including resurgence, renewal, and extinction bursts (Briggs et al., 2018; Falligant et al., 2021, 2022, 2024; Haney et al., 2022; Kranak & Falligant, 2021; Laureano et al., 2024; Mitteer et al., 2022; Muething et al., 2020; Muething, Cariveau, et al., 2024; Muething et al., 2021; Muething, Ritchey, et al., 2024). For example, researchers have manipulated reinforcer dimensions (Fleck et al., 2023; Weinsztok & DeLeon, 2022), arranged unique training histories for target and alternative responses (Lambert et al., 2015), and taught multiple alternative responses (Diaz‐Salvat et al., 2020) in an effort to reduce treatment relapse. Others have incorporated the discriminative stimuli of a multiple schedule (Fisher et al., 2020; Fuhrman et al., 2016) or made modifications based on quantitative models of behavior (e.g., Fisher, Greer, Craig, et al., 2018) to increase the durability of treatments (see Kimball et al., 2023, for review).

One important development in this area of research has been the availability of quantitative models of behavior, like behavioral momentum theory (BMT; Shahan & Sweeney, 2011; see Smith & Greer, 2023, for an overview and Greer et al., 2016, for a tutorial) and the temporally weighted matching law (also known as resurgence as choice; Greer & Shahan, 2019; Shahan, 2022; Shahan et al., 2020; Shahan & Craig, 2017), in understanding treatment relapse. Quantitative models of behavior provide applied behavior analysts with testable predictions regarding clinically relevant behavioral phenomena (Fisher et al., 2022, 2023; Greer et al., 2022; Jarmolowicz et al., 2021) and suggest a range of variables for improving long‐term maintenance of common interventions.

BMT takes analogies from Newton's laws of motion wherein an external disruptor (e.g., extinction) can change the “velocity” (i.e., response rate) of a behavior depending on that behavior's “mass,” with velocity largely determined by operant contingencies (e.g., reinforcement schedule) and mass by Pavlovian pairings between the reinforcer and context (Greer et al., 2016; Nevin & Grace, 2000; Nevin et al., 1983; Nevin & Shahan, 2011). Behavioral momentum can be assessed during a treatment challenge (e.g., transitioning from reinforcement to extinction) by examining the response's persistence in the face of that disruption. Shahan and Sweeney (2011) extended BMT by providing equations that could help describe resurgence. *Resurgence* is said to occur when a previously reinforced response (e.g., destructive behavior) recurs due to a worsening in current reinforcement conditions (Cleland et al., 2001; Lattal et al., 2017). This adaptation of BMT to resurgence allows researchers and practitioners to consider specific independent variables informed by the equations' parameters that might help alleviate resurgence. Specifically, predictions based on BMT suggest that leaner reinforcement schedules in baseline and treatment as well as a longer treatment duration should reduce resurgence of destructive behavior when treatments like functional communication training (Carr & Durand, 1985) encounter a treatment challenge, such as prolonged extinction of communication responses.^1^

We examined these BMT‐informed treatment refinements across four studies (Fisher, Greer, Fuhrman, et al., 2018; Fisher et al., 2019; Greer et al., 2020; Irwin Helvey et al., 2023) in which we arranged nearly identical experimental procedures and enrolled a similar clinical sample of outpatients referred for the assessment and treatment of destructive behavior across each study. Participants experienced three phases consisting of reinforcement for destructive behavior in baseline (Phase 1); treatment with functional communication training, which arranged reinforcement for functional communication responses and extinction for destructive behavior (Phase 2); and an extinction challenge (i.e., resurgence test) involving termination of reinforcement for functional communication responses (Phase 3). A multielement design allowed the investigators to program the independent variable predicted by BMT to mitigate resurgence to be present in one of two conditions. The two conditions could be described as *test* and *control* in which the programmed independent variable was or was not present, respectively. Lower levels of destructive behavior in the test condition than in the control condition during Phase 3 were interpreted as evidence supporting the BMT's ordinal predictions.

In the first experiment (Fisher, Greer, Fuhrman, et al., 2018), the independent variable programmed in the test condition combined three consequent‐based refinements to functional communication training, all predicted by BMT to mitigate resurgence. The experimenters programmed leaner schedules of reinforcement in the test condition (relative to the control condition) for destructive behavior in Phase 1 and for alternative behavior in Phase 2, and the experimenters also conducted three times the number of treatment sessions for the test condition than for the control condition during Phase 2. The experimenters evaluated this independent variable across four children diagnosed with autism spectrum disorder. In this article, we refer to these participants as the *combined‐Tx group*.

The three subsequent experiments isolated each of the tactics from the first experiment. Fisher et al. (2019) isolated the effects of a leaner schedule of reinforcement for destructive behavior in Phase 1 with seven children diagnosed with autism spectrum disorder. In the current article, we refer to these participants as the *target‐S* ^*r*^ *group*. Greer et al. (2020) isolated the effects of programming a longer duration of exposure to treatment in Phase 2 with six children, the majority of whom had a diagnosis of autism spectrum disorder. We refer to these participants as the *Tx‐duration group*. Irwin Helvey et al. (2023) isolated the effects of programming a leaner schedule of reinforcement for alternative behavior in Phase 2 with four children, most of whom had a diagnosis of autism spectrum disorder. We refer to these participants as the *alt‐S* ^*r*^ *group*.

Describing the findings of each experiment across separate articles may have obscured the integrated approach used across experiments, which, in turn, precluded a broader understanding of the relative effects of the resurgence‐mitigation tactics. The purpose of the present study was to address this limitation by compiling all the data sets from these studies and examining the relative efficacy of each tactic at mitigating resurgence. We included three additional data sets since the initial publications and analyses between raw response rates and proportion‐of‐baseline transformations. Overall, this approach may facilitate a clearer interpretation of which tactics appear most fruitful for further translation or implementation more broadly relative to the four separate articles published across 5 years.

## METHOD

### Participants and settings

Data sets from a total of 24 children (18 males, 6 females) were included in the current study. The original data for most of these participants can be found in Fisher, Greer, Fuhrman, et al. (2018), Fisher et al. (2019), Greer et al. (2020), and Irwin Helvey et al. (2023). Three of these children (Sean, David, and Dalinar) completed their participation following the publications. Table 1 displays participant demographics. Caregivers provided informed consent for enrollment of participants in an institutional‐review‐board‐approved research study, which described all four potential studies to which their children might be randomly assigned. Research occurred at three university‐affiliated clinics for the assessment and treatment of severe destructive behavior. Therapy rooms were approximately 3 × 3 m and contained (a) padding on the floors and walls to minimize risk of self‐injurious behavior, (b) one‐way observation mirrors for unobtrusive data collection, and (c) two‐way intercom systems for therapists and data collectors to communicate. A team of doctoral‐level board‐certified behavior analysts oversaw each participant's research study, reviewed the research data as a group at least twice a week, and routinely observed the research sessions.

**TABLE 1:** Participant characteristics.

Independent variable	Participant	Age	Gender	ASD	Race or ethnicity	Function(s) targeted
Target S^r^ Rate (Fisher et al., 2019)	Marcus	4	Male	Yes	Asian/Unknown	Tangible
	Owen	15	Male	Yes	Unknown/Hispanic	Escape
	Harvey	7	Male	Yes	White/Unknown	Escape
	Ryan	8	Male	Yes	Asian/Unknown	Tangible
	Tamra	7	Female	Yes	White/Unknown	Escape
	Sean^a^	6	Male	Yes	Unknown/Hispanic	Tangible
	Ace^b^	6	Male	Yes	Unknown/Hispanic	Tangible
	Bernie^b^	10	Male	Yes	White/Unknown	Tangible
Alternative S^r^ Rate (Irwin Helvey et al., 2023)	Tali	12	Female	No	White/Unknown	Tangible
	Zavier	6	Male	Yes	White/Unknown	Tangible
	Kendall	5	Female	Yes	Unknown/Hispanic	Tangible
	Liam^b^	7	Male	No	White/Unknown	Tangible
Treatment Duration (Greer et al., 2020)	Oliver	8	Male	Yes	White/Unknown	Tangible
	Samuel	12	Male	Yes	White/Unknown	Tangible + Attention
	Nancy	11	Female	Yes	Unknown/Hispanic	Tangible + Attention
	Bentley	9	Male	Yes	Black or African American/Unknown	Tangible + Attention
	Esther	4	Female	No	White/Unknown	Tangible + Attention
	Javis	8	Male	Yes	Black or African American/Unknown	Tangible
Combined Approach (Fisher, Greer, Fuhrman, et al., 2018)	Erica	16	Female	Yes	White/Unknown	Tangible
	Corey	3	Male	Yes	White/Unknown	Attention
	Jaden	8	Male	Yes	Black or African American/Non‐Hispanic	Tangible
	Derek	7	Male	Yes	White/Unknown	Tangible
	David^a^	14	Male	No	White/Non‐Hispanic	Escape + Tangible
	Dalinar^a^	7	Male	Yes	White/Non‐Hispanic	Escape + Tangible

*Note*: S^r^ = reinforcement.

^a^ New data set following publication.

^b^ Data excluded from current analysis.

### Response measurement, interobserver agreement, and procedural fidelity

Across all studies, data collectors recorded the frequency of destructive behavior (e.g., aggression, self‐injurious behavior) using BDataPro (Bullock et al., 2017), which automatically converted the frequency of destructive behavior to a rate measure (responses per minute). A second, independent observer scored data on at least 26% of sessions to compute exact agreement, with average interobserver agreement coefficients of 82% or higher across the studies. Because procedural fidelity of the independent variables usually involved the extent to which obtained reinforcer deliveries matched programmed reinforcer deliveries, most of the articles provide explicit summaries of reinforcer deliveries, which all approximated the programmed levels of the independent variables. Please see Fisher, Greer, Fuhrman, et al. (2018), Fisher et al. (2019), Greer et al. (2020), and Irwin Helvey et al. (2023) for specific response definitions, interobserver agreement data, and obtained‐reinforcement details.

### General study procedures

The four studies involved highly similar procedures and evaluated resurgence within a three‐phase arrangement that embedded a multielement design consisting of a test and control condition. Each test and control condition occurred within a distinct context (e.g., a room with yellow lights and yellow poster boards; a room with blue lights and blue poster boards) to facilitate discrimination between the conditions.

#### Baseline

During baseline, destructive behavior produced the maintaining reinforcer (identified by a preexperimental functional analysis) on a variable‐interval (VI) schedule using the constant‐probability distribution described by Fleshler and Hoffman (1962). Generally, reinforcement consisted of 20 s of access to the maintaining reinforcer. The number of intervals depended on the assigned VI value and reinforcement duration (e.g., we programmed ten 20‐s reinforcement deliveries for a 10‐min session that involved a VI 40‐s schedule). One notable exception was Experiment 2 in Fisher et al. (2019), which involved a fixed‐ratio 1 schedule during baseline. When evaluating the effects of target reinforcement rate (target‐S^r^ group) and the combined approach (combined‐Tx group), one condition involved a lean schedule of reinforcement (e.g., VI 90 s) and the other used a dense schedule of reinforcement (e.g., VI 20 s). Experimenters individualized VI schedules for each participant based on latencies to destructive behavior and results of a progressive‐interval assessment (see Fisher, Greer, Fuhrman, et al., 2018, for a description). Across studies, baseline continued for at least five sessions and until destructive behavior met the following criteria in each condition: (a) the trend was flat or increasing and (b) the standard deviation was no more than 50% of the mean.

#### Treatment

Treatment (i.e., functional communication training) involved differential reinforcement of communication responses and extinction following destructive behavior. Communication responses produced reinforcement on a VI schedule, again using the Fleshler and Hoffman (1962) constant‐probability distribution, for approximately 20 s. During the experiments involving alternative reinforcement rate and the combined approach, one condition incorporated a lean schedule of reinforcement (e.g., VI 72 s) and the other condition involved a dense schedule of reinforcement (e.g., VI 4 s). Again, experimenters individualized these VI values for each participant. During experiments investigating treatment dose (Tx‐duration group) and the combined independent variables (combined‐Tx group), participants experienced the test condition three times as often as the control condition. Treatment ended following a requisite number of sessions (depending on the study question) and after obtaining a clinically significant decrease (e.g., 85% or greater) from the respective baseline's mean in both conditions.

#### Extinction challenge

The last phase tested for resurgence. Therapists programmed extinction for both destructive behavior and communication responses, which might approximate a situation that could engender treatment relapse (i.e., a treatment challenge) outside of the clinic (e.g., when their tablet battery is depleted and reinforcement is unavailable for a prolonged period). In the initial study evaluating the combined approach (Fisher, Greer, Fuhrman, et al., 2018), therapists also programmed a variable‐time (VT) 200‐s reinforcement delivery to decrease the discriminability of transitioning from functional communication training to extinction. However, we removed the VT reinforcement, as such response‐independent deliveries may have contributed to another form of relapse known as reinstatement (for a primer, see Kranak & Falligant, 2023). Across studies, the extinction challenge continued until destructive behavior decreased by 85% or greater from each condition's baseline for at least two consecutive sessions or until conducting a requisite number of sessions (e.g., 10 sessions in each condition). As noted below, some studies involved many more extinction‐challenge sessions per condition (e.g., 10 sessions) than others (e.g., 3 sessions), and later sessions in the extinction challenge may show diminished resurgence due to continued exposure to extinction. Therefore, all analyses in this article will involve the first three sessions per condition within the extinction challenge.

### Data transformation

As some of the data sets involved differential rates of reinforcement during baseline (e.g., Fisher, Greer, Fuhrman, et al., 2018 [combined‐Tx group]; Fisher et al., 2019 [target‐S^r^ group]) and because some participants exhibited higher rates of destructive behavior than others, we converted each participant's extinction‐challenge data into a proportion of baseline. Specifically, we divided each session's value for destructive behavior per minute by the average destructive behavior per minute in the participant's final five baseline sessions. The closer the value was to 1.0, the greater the responding approximated baseline. Values greater than 1.0 indicated that resurgence exceeded baseline levels of destructive behavior. For example, if a participant displayed 2 responses per minute during the extinction challenge in the condition associated with lean reinforcement of target behavior and 4 responses per minute during the extinction challenge in the condition associated with dense reinforcement of target behavior, we divided each of those values by the average of the respective condition's baseline. Suppose that the average baseline values were 8 and 4 responses per minute for the lean and dense conditions, respectively. This would result in a proportion‐of‐baseline value of 0.25 (2 ÷ 8) for the lean condition and 1.0 (4 ÷ 4) for the dense condition. Thus, despite destructive behavior in the extinction challenge occurring twice as often in the condition associated with dense reinforcement, the level of resurgence in relation to that respective baseline would be substantially higher.

### Data analysis plan

#### Data exclusion

Because we were interested in determining the relative efficacy of each independent variable for mitigating resurgence, we opted to exclude cases in which we did not observe meaningful levels of resurgence in either condition (i.e., test and control). During treatment (Phase 2), functional communication training ended following a clinically relevant reduction in destructive behavior (i.e., an 85% or greater reduction from baseline levels). If this clinically relevant reduction maintained in both conditions during the extinction challenge (Phase 3), we excluded the data set from the analysis. This helped to ensure that our analysis focused only on cases with appreciable resurgence in at least one condition and resulted in excluding three data sets (Ace, Bernie, Liam) from the analysis, each of which contained three data points per test and control condition in the extinction challenge that maintained at or below the reduction criteria (i.e., minimal to no resurgence). Data and figures for these excluded data are available from the first author upon request.

#### Percentage reduction in resurgence

To determine the relative effect of an independent variable on the proportion‐of‐baseline responding for each data set, we computed the *percentage reduction in resurgence* for each participant. First, we computed the mean proportion‐of‐baseline value for the test condition (i.e., lean target reinforcement, lean alternative reinforcement, long treatment duration, lean target reinforcement plus lean alternative reinforcement plus long treatment duration). Then, we computed the mean proportion‐of‐baseline value for the control condition (i.e., dense target reinforcement, dense alternative reinforcement, short treatment duration, dense target reinforcement plus dense alternative reinforcement plus short treatment duration). As noted earlier, some data sets involved many more extinction‐challenge sessions per condition than others, so we analyzed the first three extinction‐challenge sessions per condition for each data set. Then, we divided the mean for the test condition by that for the control condition and subtracted the quotient from 1. This number was then converted to a percentage. For example, the means for Corey from Fisher, Greer, Fuhrman, et al. (2018) were 0.11 and 0.59 for the test and control conditions, respectively. This constituted approximately an 81.36% reduction ([1 − (0.11 ÷ 0.59)] × 100) in resurgence with the combined approach relative to the control condition. Accounting for the proportion‐of‐baseline levels in the participants' control conditions with the calculation for percentage reduction allowed us to standardize the overall mitigation effect across participants and independent variables. As described in the next section, we later completed the same calculation for two treatment groups in particular but with the mean of raw response rates rather than proportion‐of‐baseline values.

After calculating the percentage reduction in proportion‐of‐baseline values for each of the 21 included data sets, we categorized the data sets into groups according to the independent variable tested: (a) target‐S^r^ group (Fisher et al., 2019), (b) alt‐S^r^ group (Irwin Helvey et al., 2023), (c) Tx‐duration group (Greer et al., 2020), and (d) the combined‐Tx group (Fisher, Greer, Fuhrman, et al., 2018). Due to the low sample size in each group, particularly alt‐S^r^ following data exclusion, we did not conduct statistical testing to determine whether the obtained differences were statistically significant.

#### Comparison of proportion‐of‐baseline and response‐rate data

After identifying the groups that produced the most robust and consistent resurgence‐mitigation effects within our percentage‐of‐reduction analysis, we sought to determine whether those mitigation findings would remain clinically significant when analyzing extinction‐challenge data as raw response rates as opposed to proportion‐of‐baseline values. Accounting for differing baseline rates of destructive behavior with a proportion‐of‐baseline transformation is important conceptually because it allows one to assess the amount of response recovery according to pretreatment levels. However, a mitigation strategy that produces high rates of destructive behavior during a worsening of alternative reinforcement (e.g., the extinction challenge), even if it is not as pronounced as the higher baseline response rate, can make that treatment option less pragmatic for clinical practice. Furthermore, what appeared to be appreciable differences in resurgence during the extinction challenge could, in fact, be a function of differential baseline response rates (e.g., as programmed in the test and control conditions of the target‐S^r^ and combined‐Tx groups). Therefore, we recalculated percentage‐of‐reduction data using raw response rates during the extinction challenge and focused our analysis on the two treatment groups that had the most promising mitigation effects initially (i.e., target‐S^r^ and combined‐Tx groups).

## RESULTS

### Proportion‐of‐baseline analysis

**FIGURE 1:**
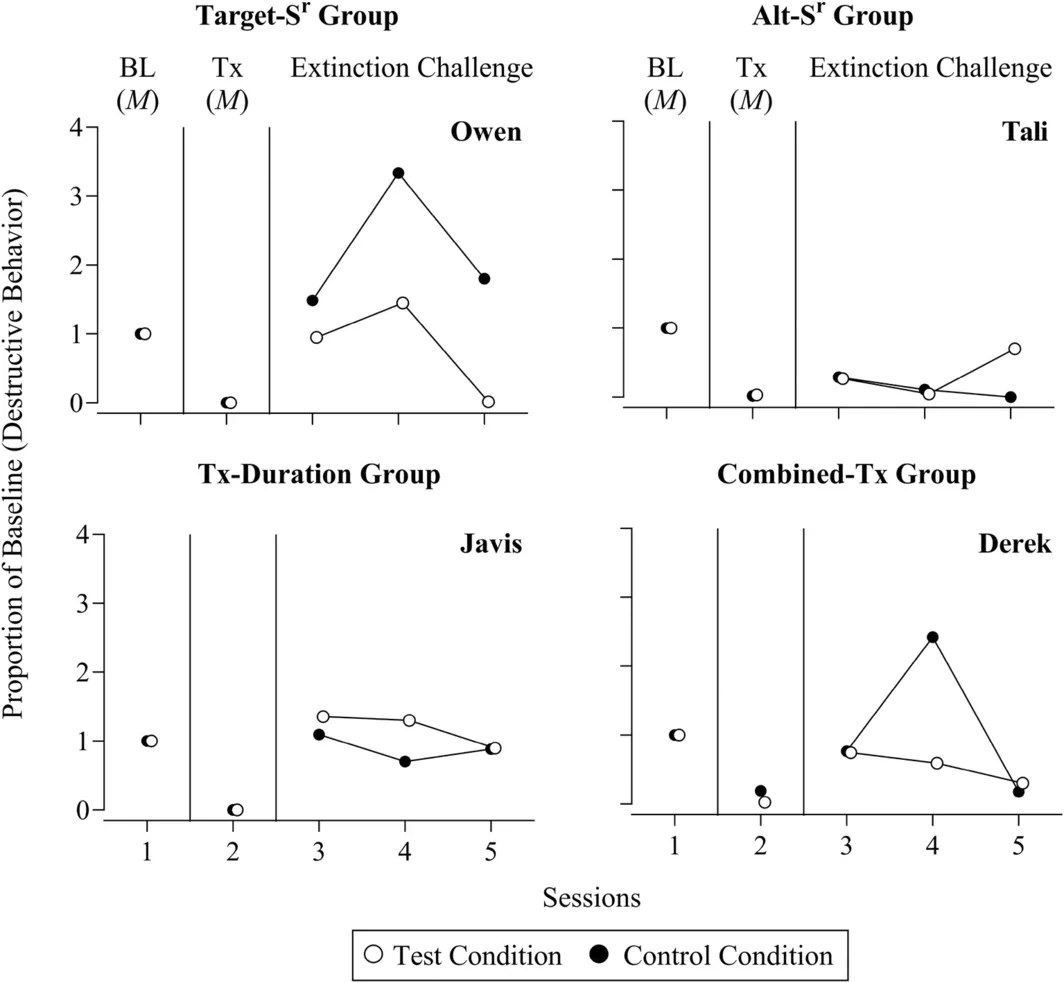
Representative data from the four analyzed studies. Representative data depicted as proportion‐of‐baseline responding, where values closer to 1 approximate baseline (BL) rates of destructive behavior. Values above 1 indicate that resurgence exceeded BL rates. Experimenters conducted sessions quasirandomly (no more than two consecutive sessions of the same condition) within a multielement design; however, data are displayed stacked with test data points nudged slightly to the right for ease of visual inspection across figures. S^r^ = reinforcement; Alt = alternative; Tx = treatment; and *M* = mean.

Figure 1 displays representative data from each of the four groups when transforming raw response rates into proportion‐of‐baseline values. Specifically, these data sets had proportion‐of‐baseline reductions in resurgence closest to the median percentage‐of‐reduction calculation for each group (i.e., resurgence reduction in the test condition relative to the control condition in the first three extinction‐challenge sessions), described in the next paragraph. Because the results of interest involved the proportion‐of‐baseline responding during the test and control conditions during the extinction challenge in Phase 3, we present the mean for the final five baseline sessions (Phase 1), mean for the final two treatment sessions (Phase 2), and first three extinction‐challenge sessions (Phase 3). The first data point representing baseline for each condition will always be a proportion of 1 because it involves dividing the mean of baseline responding by itself (e.g., 4.83 ÷ 4.83 = 1). The second data point, representing the average proportion‐of‐baseline responding during the final treatment sessions, showed that all interventions involving functional communication training, irrespective of reinforcement rate or dose, effectively reduced destructive behavior from baseline. The final phase displays responding during the critical test periods for resurgence.

As displayed in Figure 1, the representative data sets from each of the four groups showed clinically significant levels of resurgence during Phase 3, nearing or exceeding the mean baseline levels of destructive behavior in at least one session for each representative participant. At least one session in each condition demonstrated resurgence in 83.33% (5/6) of cases in the target‐S^r^ group, 66.67% (2/3) of cases in the alt‐S^r^ group, 66.67% (4/6) of cases in the Tx‐duration group, and 83.33% (5/6) of cases in the combined‐Tx group. Two of the representative participants showed significantly lower levels of resurgence in the test condition relative to the control condition. These were Owen (top left), whose data set is representative of the target‐S^r^ group, and Derek (bottom right), whose data set is representative of the combined‐Tx group.

**FIGURE 2:**
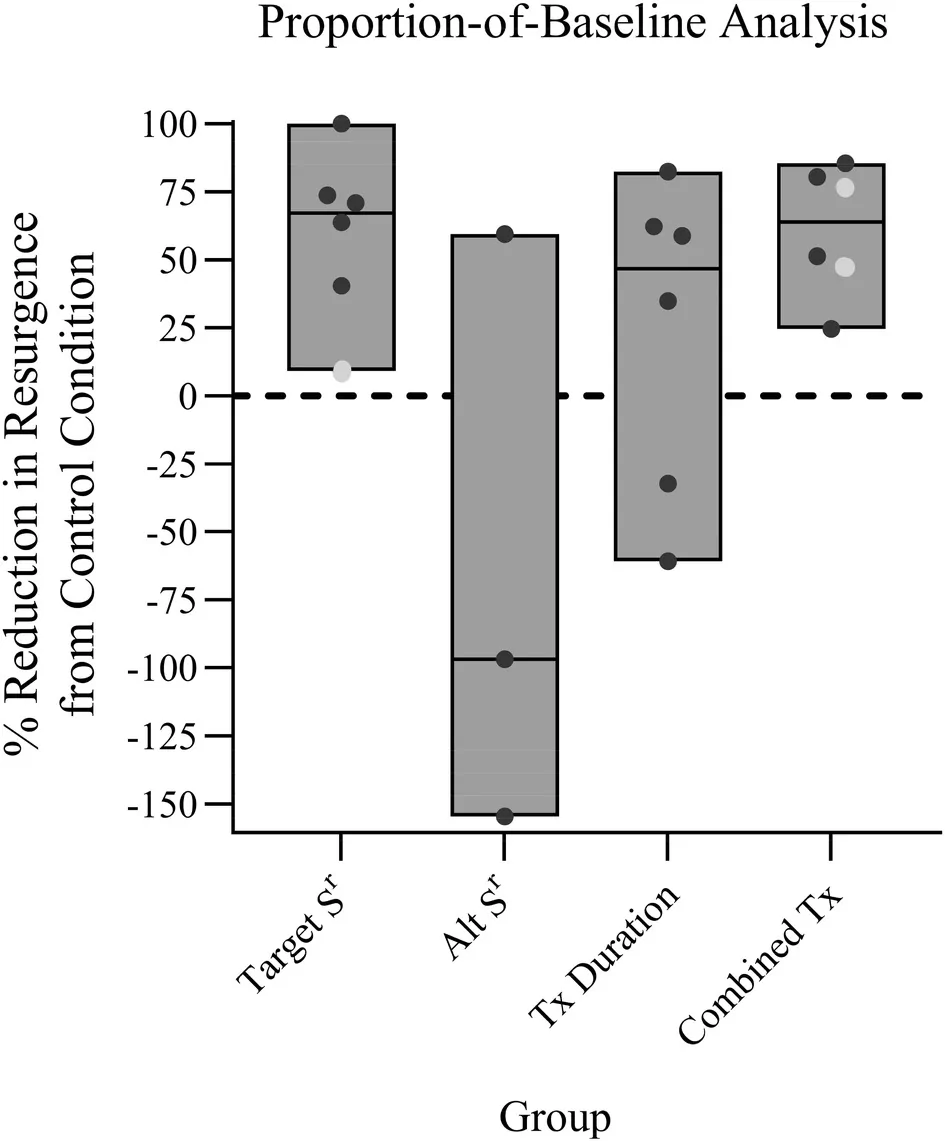
Percentage reduction in resurgence during the test condition relative to the control condition (proportion of baseline). Bars depict the range of percentage‐reduction data, with solid horizontal lines showing the median and dark circles showing each participant's data. Percentages in this figure were derived from proportion‐of‐baseline values. Light gray data points indicate new data sets added after publication. The dashed horizontal line represents no reduction (0%) in resurgence in the test condition relative to the control condition. S^r^ = reinforcement; Alt = alternative; Tx = treatment.

Figure 2 shows the medians, ranges, and individual data points for each included participant across the four experimental groups, again in relation to the proportion‐of‐baseline values during the extinction challenge. The scores indicate the percentage reduction in resurgence in the test condition relative to the control condition when accounting for the average proportion‐of‐baseline value in the control condition relative to the average proportion‐of‐baseline value in the test condition. Scores above zero indicate that the test condition reduced the baseline proportional level of resurgence relative to the control condition; scores below zero indicate that the test condition increased the baseline proportional level of resurgence relative to the control condition.

As shown by the data for the first bar on the left of Figure 2, lowering the rate of reinforcement for the target response in baseline in the test condition for target‐S^r^ group reduced the baseline proportional level of resurgence relative to the control condition for all six participants (all scores above zero; *Mdn* = 67.14%).

The second bar from the left in Figure 2 shows that lowering the rate of reinforcement in the test condition for the alt‐S^r^ group during treatment reduced the baseline proportional level of resurgence somewhat for one participant (59.30%) but increased it considerably for the other two (−96.95% and −154.57%).

The third bar from the left in Figure 2 shows that extending the duration of exposure to reinforcement of the alternative response during treatment in the test condition of the Tx‐duration group reduced the baseline proportional level of resurgence relative to the control condition for four participants (i.e., 34.61, 58.69, 62.13, and 82.30%). However, resurgence increased in the test condition relative to the control condition for the other two participants (i.e., −32.29%; −60.81%).

The bar on the right of Figure 2 shows the effects of combining all three BMT‐informed mitigation techniques within the combined‐Tx group (i.e., reducing the rate of reinforcement for the target response in baseline, reducing the rate of reinforcement for the alternative response during treatment, and extending the duration of exposure to treatment). As shown, this combined approach reduced the baseline proportional level of resurgence in the test condition relative to the control condition for all six participants (all scores above zero; *Mdn* = 63.88%).

To summarize, our analysis of proportion‐of‐baseline responding indicated resurgence mitigation in 6 of 6 (100%) target‐S^r^ participants, 1 of 3 (33.33%) alt‐S^r^ participants, 4 of 6 (66.67%) Tx‐duration participants, and 6 of 6 (100%) combined‐Tx participants. The most robust mitigation effects (over 60% reduction in resurgence) occurred in the target‐S^r^ and combined‐Tx groups.

### Comparison with raw response rates

**FIGURE 3:**
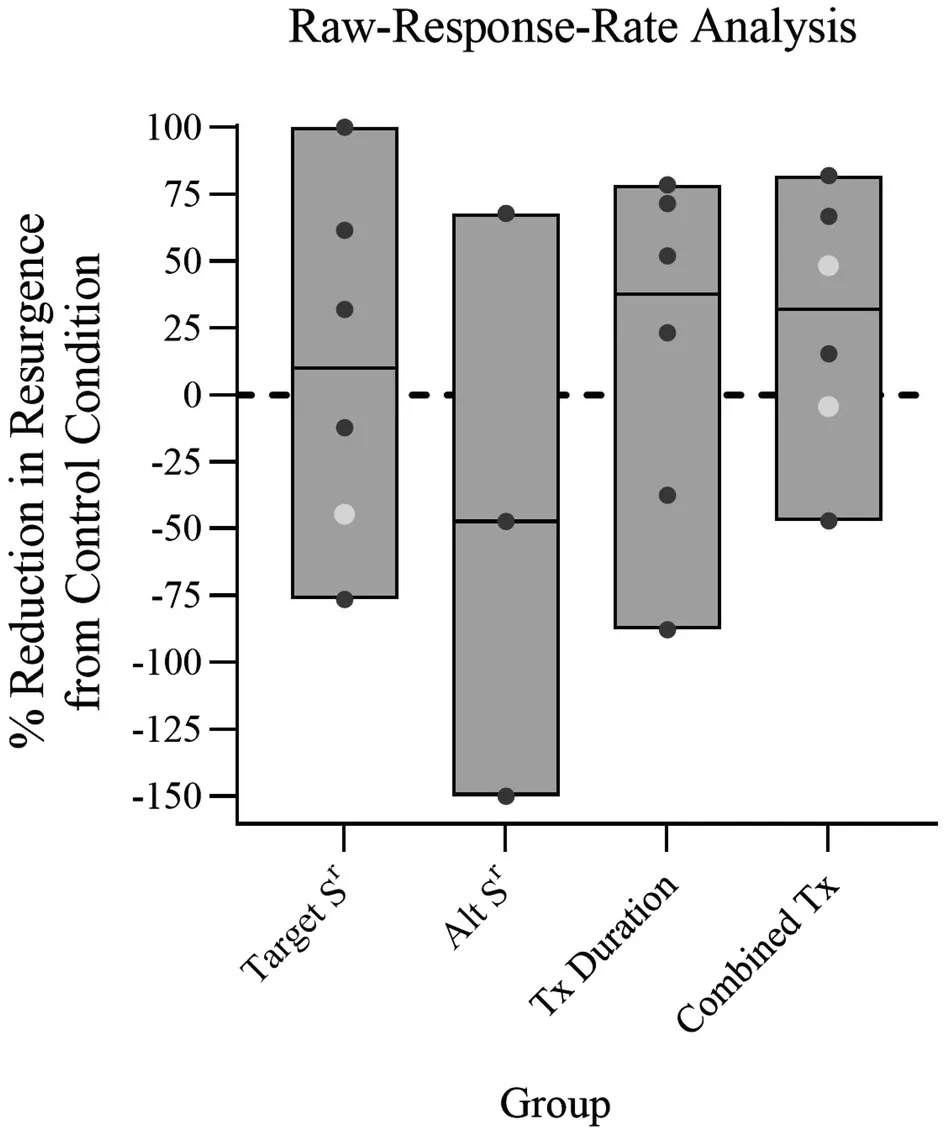
Percentage reduction in resurgence during the test condition relative to the control condition (raw response rates). Bars depict the range of percentage‐reduction data, with solid horizontal lines showing the median and dark circles showing each participant's data. Percentages in this figure were derived from raw‐response‐rate values. Light gray data points indicate new data sets added after publication. The dashed horizontal line represents no reduction (0%) in resurgence in the test condition relative to the control condition. S^r^ = reinforcement; Alt = alternative; Tx = treatment.

Figure 3 is identical to Figure 2 except that the percentage‐of‐reduction calculations were derived from differences in raw response rates during the extinction challenge. Generally, the alt‐S^r^ and Tx‐duration groups remained similar in this analysis because there was no difference between baseline reinforcer rates during test and control conditions in a way that would likely produce differential baseline response rates and warrant a proportion‐of‐baseline transformation. The alt‐S^r^ median percentage‐of‐reduction score increased by 48.59% but still produced a mitigation effect for only one participant. The Tx‐duration median decreased by 9.16%, again reducing resurgence for four participants but increasing resurgence for the other two participants.

As expected, the target‐S^r^ and combined‐Tx groups were the most affected because they involved response and reinforcer differences during baseline. The target‐S^r^ group's median percentage reduction decreased by 57.31% (i.e., *Mdn =* 9.84%), and the combined‐Tx group's median was reduced by 32.10% (i.e., *Mdn =* 31.78%), although the treatment refinements continued to produce positive (albeit diminished) mitigation effects. Previously, the percentage‐of‐reduction calculation based on proportion‐of‐baseline values had resulted in a resurgence‐mitigation effect for all six participants in each group. When analyzing raw response rates, the target‐S^r^ group showed mitigation effects for half of participants (i.e., 31.91, 61.54, 100%) but increases in resurgence for the other three (i.e., −12.24, −44.68, −76.47%). The combined‐Tx group fared better, with four instances of resurgence mitigation (i.e., 15.38, 48.17, 66.67, 81.82%) but two participants displaying more resurgence in the test condition (i.e., −4.49% and −47.15%).

In summary, the analysis of raw response rates indicated resurgence mitigation in 3 of 6 (50%) target‐S^r^ participants, 1 of 3 (33.33%) alt‐S^r^ participants, 4 of 6 (66.67%) Tx‐duration participants, and 4 of 6 (66.67%) combined‐Tx participants. When mitigation occurred, the median reductions were less robust than those in the proportion‐of‐baseline analysis.

**FIGURE 4:**
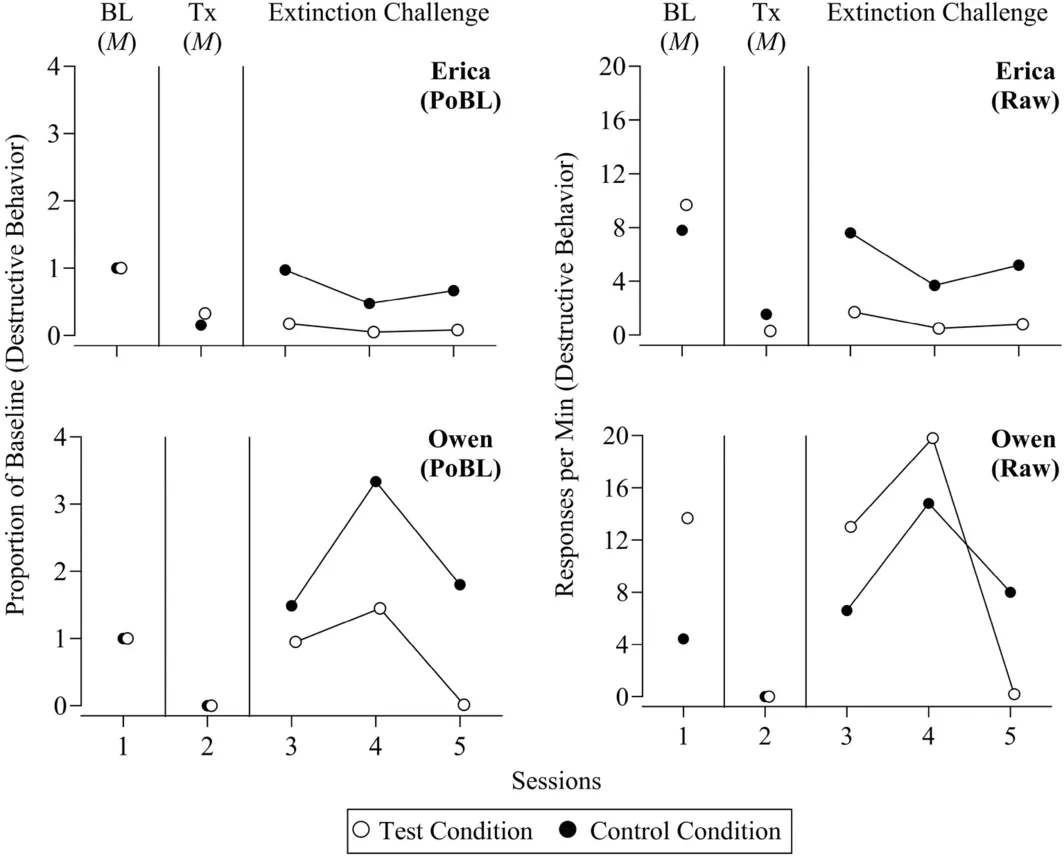
Representative comparisons of proportion of baseline (PoBL) and raw response rates. Representative data sets in which the general level of differentiation between test and control conditions during the extinction challenge correspond (Erica, top panels) or differ substantially (Owen, bottom panels) between PoBL transformation and raw response rates. BL = baseline; Tx = treatment; *M* = mean.

Figure 4 helps illustrate what promising mitigation effects seen in proportion‐of‐baseline transformations might look like in practice. The top two graph panels show the participant Erica's data as a proportion of baseline (left) or raw response rates (right). In Erica's case, the findings are about the same. As shown in the top‐left panel, the treatment refinements resulted in a reduction in destructive behavior when accounting for slightly higher baseline rates of destructive behavior in the test condition (i.e., proportion‐of‐baseline transformation) and, as shown in the top‐right panel, substantially less destructive behavior during the extinction challenge (1 response per minute versus 5.50 responses per minute). Owen (bottom two panels) is a participant for whom the treatment refinements in the test condition mitigated resurgence when accounting for baseline response rates (bottom‐left graph), but this effect was not as apparent when examining raw response rates (bottom‐right graph). In fact, the interpretation reversed for Owen's data set when examining raw response rates in that more destructive behavior occurred in the test condition (*M* = 11 responses per minute) than in the control condition (*M* = 9.80 responses per minute).

## DISCUSSION

The major findings of the current study were that when analyzing resurgence during the extinction challenge as a proportion of baseline, (a) lowering the rate of reinforcement for the target response during baseline mitigated resurgence for 6 of 6 participants, (b) lowering the rate of reinforcement for the alternative response during treatment reduced resurgence for only 1 of 3 participants, (c) extending the duration of treatment mitigated resurgence for 4 of 6 participants, and (d) combining these three resurgence‐mitigation strategies reduced resurgence for 6 of 6 participants. The combined‐Tx group produced the most consistent and robust reductions in resurgence. However, when analyzing raw response rates during the extinction challenge, the independent variables in the target‐S^r^ and combined‐Tx groups did not generate as robust and consistent mitigation, with reductions in resurgence for only 3 of 6 participants and 4 of 6 participants, respectively, and smaller values for percentage of reduction in resurgence relative to the initial proportion‐of‐baseline analysis.

These results and their implications should be considered along with the limitations of the initial experiments and the method of the current analysis. First, the sample size was small and groups did not have equal numbers of participants. This limitation is particularly germane to the alt‐S^r^ group that consisted of only three participants. If we had recruited additional participants in the alt‐S^r^ group, they could have shown response patterns akin to the one participant who experienced a 59.30% resurgence reduction following lower alternative reinforcement in the treatment phase. Second, because the source experiments were translational studies designed to control for potential confounds (e.g., using VI reinforcement schedules to better monitor reinforcer rates across conditions), the outcomes of our analysis may not correspond to typical clinical practice. For example, functional communication training might involve strategies like reinforcement schedule thinning (Hagopian et al., 2011) or reinforcer quality manipulations to favor alternative responses and reduce resurgence (e.g., Fleck et al., 2023). Evaluating the independent variables from this study with more participants and interventions closely aligned to clinical practice is essential to determining the generality of the findings. Third, although we believe that our percentage‐of‐reduction calculation and levels of analysis were appropriate, future research might be able to detect other compelling relations via reanalysis (e.g., examining within‐session responding) or novel data transformation. Despite the potential limitations, the sections below detail how our preliminary findings can be interpreted as they relate to the BMT framework, extant resurgence literature, and clinical practice.

### Relevance to basic research and conceptual understanding of resurgence

BMT posits that operant contingencies determine the rate of the target response during baseline but Pavlovian pairings between the stimulus context and the maintaining reinforcer determine its persistence once operant reinforcement is withdrawn during Phases 2 and 3. The first two mitigation strategies involved reducing the rate of reinforcement (and thereby the rate of Pavlovian pairings) for (a) the target response during baseline in the target‐S^r^ group and (b) the alternative response during treatment in the alt‐S^r^ group, respectively. It is important to note that BMT predicts that Pavlovian pairings between the stimulus context and the maintaining reinforcer increase the persistence of the target response during Phase 3 regardless of whether the reinforcer is delivered contingent on the target response during baseline (Phase 1) or the alternative response during treatment (Phase 2; Nevin et al., 1990). Whereas BMT predicts that differential‐reinforcement treatments like functional communication training suppress the target response while treatment contingencies are in effect (during Phase 2), it also predicts that the Pavlovian pairings resulting from those treatment contingencies increase the probability of resurgence once the treatment contingencies are withdrawn.

The lower levels of resurgence in the test condition relative to the control condition for the target‐S^r^ group are consistent with the predictions of BMT. Although this topic has been less investigated in humans (e.g., see the human‐operant experiment in Kuroda et al., 2016), the findings correspond with nonhuman animal studies such as Podlesnik and Shahan (2009), which found that resurgence was greater in the presence of stimuli associated with richer baseline reinforcement. However, we observed roughly equivalent levels of resurgence for the test and control conditions in the alt‐S^r^ group irrespective of the proportion‐of‐baseline transformation. These results are inconsistent with BMT's predictions and create an increasingly mixed literature related to higher or lower rates of alternative reinforcement (Craig & Shahan, 2016; Fujimaki et al., 2015; Winterbauer & Bouton, 2010; for an applied example, see Nevin et al., 2016). The current results call into question the BMT proposition that the strength or persistence of a target response during an extinction challenge is a function of the rate of Pavlovian pairings between the stimulus context and the maintaining reinforcer (for additional discussions of this conceptual issue, see Fisher et al., 2022; Shahan & Craig, 2017).

BMT also posits that the passage of time with alternative reinforcement plays a critical role in later resurgence, with longer durations assumed to mitigate resurgence relative to shorter durations (Shahan & Sweeney, 2011). Previous studies have shown a relation between treatment duration and resurgence (e.g., some improvements in resurgence at longer durations; Shahan et al., 2020; Smith & Greer, 2022), whereas others have found minimal to no effect (e.g., Trask et al., 2018; Winterbauer et al., 2013). We observed roughly equivalent levels of resurgence for the test and control conditions in the Tx‐duration group irrespective of data transformation. Greer et al. (2023) conducted a follow‐up study with a considerably wider range of treatment durations and still found no consistent effect of treatment duration on resurgence. These experimental findings correspond with a recent retrospective controlled case series analysis by a separate research lab (Laureano et al., 2024) that found a weak, nonsignificant correlation between time spent in treatment (i.e., minutes spent reinforcing participant alternative behavior) and resurgence magnitude (computed as proportion of baseline) for 40 analyzed cases. Overall, BMT's predictions for treatment duration do not appear overly helpful in mitigating resurgence of clinically significant behavior at this time.

Although the combined‐Tx group showed promising mitigation, these effects should not be interpreted as supporting all three of the hypothesized independent variables derived from BMT or that the combined treatment is the “best treatment” per se. To our knowledge, no other basic, translational, or applied study has examined the effects of combining lean reinforcement in Phases 1 and 2 with an extended treatment duration in Phase 2 as was done in the combined‐Tx group. Leaner reinforcement in baseline by itself may have resulted in the promising effects observed during the combined‐Tx group. It may be that alternative reinforcement rate, treatment duration, or both had a minimal effect in isolation but an additive effect when blended within the combined approach. Examining this possibility would require additional analyses with multiple permutations (e.g., differing rates of alternative reinforcement with differing rates of treatment duration and lean target reinforcement). Given the required sample size for such a comparison and other compelling treatment refinements for applied work, such an endeavor might be best addressed with basic or translational arrangements.

Ultimately, our conclusions suggest that BMT—although being an important and enduring theory—represents an imperfect framework for understanding and mitigating resurgence (for larger discussions on this topic, see Craig & Shahan, 2016; Fisher et al., 2022; Greer & Shahan, 2019; Shahan & Craig, 2017). We encourage researchers and clinicians to look to alternative theories of resurgence, like the temporally weighted matching law (formerly described as resurgence as choice; e.g., Shahan, 2022), which may provide new avenues for better predicting and controlling resurgence.

### Implications for applied research and clinical practice

Our data do not support deliberately arranging lengthy treatment phases (i.e., Tx‐duration group) as a way to offset later resurgence. The current practice of progressing treatment (e.g., conducting reinforcement schedule thinning) shortly after obtaining initial treatment effects does not appear to engender more resurgence during prolonged periods of extinction for communication responses. Programming leaner reinforcement schedules for communication responses at the onset of treatment (i.e., alt‐S^r^ group) did not appreciably reduce resurgence during the extinction challenge. A typical schedule‐thinning arrangement in which clinicians begin with a dense reinforcement schedule for communication responses (for overviews, see Hagopian et al., 2011; and Kranak & Brown, 2024) does not appear to worsen resurgence during extinction challenges. There may be other benefits when arranging initially lean schedules for communication responses early on in treatment, such as reaching the final reinforcement schedule more quickly (Chesbrough et al., 2024; Hagopian et al., 2004) and abating resurgence during the schedule thinning process (Strohmeier et al., 2024).

The most robust resurgence‐mitigation effects occurred in the target‐S^r^ and combined‐Tx groups. Although these results are important, clinicians must balance the potential for resurgence mitigation with the practical limitations of these refinements. In terms of the combined treatment approach, it seems premature to suggest that clinicians ought to be packaging lengthy treatment duration and lean alternative reinforcement with lean target reinforcement given the limited efficacy of those first two variables in isolation and practical challenges of programming lean alternative reinforcement for a prolonged time without progressing treatment goals.

For the other remaining independent variable—lean target reinforcement—a clinician must consider whether a leaner schedule of reinforcement that engenders a fair amount of destructive behavior in baseline is more desirable than an increased possibility of resurgence later on in treatment. In the target‐S^r^ group, the lean reinforcement schedule produced an average rate of destructive behavior ranging between 2.40 and 11.26 responses per minute and a grand mean of 6.30 responses per minute. Depending on the severity of the destructive behavior and risk of tissue damage to self or others, this modification might not outweigh the potential benefit of lessened resurgence during later treatment challenges. A clinician might consider a lean target reinforcement rate for challenging behavior that does not pose imminent harm, such as with disruptive behavior (e.g., ripping papers, vocal protests), or when providing consequences for precursor behavior. This might reduce the recurrence of resurgence while not posing immediate risks with higher rates of target behavior in baseline.

Finally, our results suggest that researchers should generally consider raw response rates for any strategy that appears promising with transformed data. It is appropriate to question whether the perceived mitigation in the target‐S^r^ and combined‐Tx groups is merely an artifact of transforming the data to a proportion of baseline. There were some raw‐response‐rate data sets in which the percentage reduction in resurgence decreased and a few in which the outcome was the opposite from the proportion‐of‐baseline calculation (e.g., more resurgence in the test condition). We still observed mitigation effects for half of the target‐S^r^ participants and two thirds of the combined‐Tx participants when analyzing raw response rates in the extinction challenge. Thus, our general findings about resurgence mitigation are probably not due to artificially low baseline response rates or post hoc data transformation exclusively. However, proportion‐of‐baseline values could obscure just how high rates of severe destructive behavior are in the test condition, even if proportion‐of‐baseline values are lower than those in the control condition. If severe aggression or self‐injury resurge and occur at moderate or high rates during the test condition of a relapse evaluation, it may not matter how much “worse” those responses are in the control condition—the client or others might be at immediate risk for injury. We are not suggesting that researchers avoid using a proportion‐of‐baseline transformation, but we encourage close examination of raw response rates during relapse or durability tests in addition to those transformed measures.

Although null findings can be a disappointment when we hope the intervention approaches will better support individuals with severe destructive behavior and their families, studies such as these can help us refocus our resources (i.e., funding priorities, time, effort) toward other promising independent variables. Applied researchers have examined alternative strategies for minimizing resurgence, such as embedding discriminative stimuli during functional communication training (Fisher et al., 2020; Fuhrman et al., 2016). This strategy has not only reduced the proportion‐of‐baseline responding in the test condition relative to the control condition within the experiments but also often generated minimal raw response rates that would be clinically acceptable during treatment of severe destructive behavior. Additionally, there are promising basic and translational studies with potential treatment refinements that have yet to be adapted with clinical populations (for a review, see Kimball et al., 2023). Thus, we encourage clinicians to remain in touch with the resurgence literature over the next several years, as there are sure to be new and compelling research projects with the possibility of altering standard‐of‐care treatments.

## AUTHOR CONTRIBUTIONS

The first author contributed to the conceptualization of the project, curated and analyzed the data, created the data visualizations, drafted portions of the initial manuscript, and contributed to revisions of the manuscript. The second author led conceptualization of the project, supervised data analysis and visualization, contributed to the initial draft of the manuscript, and assisted with manuscript revisions. The third author contributed to conceptualization of the project, supervised data analysis and visualization, drafted portions of the initial manuscript, and assisted with manuscript revisions. The fourth author assisted with data curation and analysis, contributed to initial manuscript drafts, and assisted with manuscript revisions.

## CONFLICT OF INTEREST STATEMENT

There is no conflict of interest to declare.

## ETHICS APPROVAL

Included data sets were collected at the University of Nebraska Medical Center, CSH–RUCARES, and Upstate Medical University. All studies included approval by respective institutional review boards and caregivers consented to their children participating. All procedures performed in studies involving human participants were conducted in accordance with the ethical standards of the institutional review boards and with the 1964 Helsinki declaration and its later amendments or comparable ethical standards.

## Data Availability

The data sets generated during and/or analyzed during the current study are available from the corresponding author on reasonable request.

## Footnotes

^1^
Those interested in better understanding BMT equation parameters and simulating data based on the modifications described in this article should consider the tutorial by Greer et al. (2016), which guides readers through Microsoft Excel workbook exercises.
